# Bridge Displacement Monitoring Method Based on Laser Projection-Sensing Technology

**DOI:** 10.3390/s150408444

**Published:** 2015-04-13

**Authors:** Xuefeng Zhao, Hao Liu, Yan Yu, Xiaodong Xu, Weitong Hu, Mingchu Li, Jingping Ou

**Affiliations:** 1 State Key Laboratory of Coastal and Offshore Engineering, School of Civil Engineering, Dalian University of Technology, Dalian 116000, China; E-Mails: liuhao200803308@163.com (H.L.); oujinping@hit.edu.cn (J.O.); 2 School of Electronic Science and Technique, Dalian University of Technology, Dalian 116000, China; E-Mail: yuyan@dlut.edu.cn; 3 School of Software Technology, Dalian University of Technology, Dalian 116000, China; E-Mails: xxdcan@mail.dlut.edu.cn (X.X.); huweitong420@gmail.com (W.H.); mingchul@dlut.edu.cn (M.L.); 4 School of Civil Engineering, Harbin Institute of Technology, Harbin 150000, China

**Keywords:** laser device, laser spot centroid, video, displacement monitoring

## Abstract

Bridge displacement is the most basic evaluation index of the health status of a bridge structure. The existing measurement methods for bridge displacement basically fail to realize long-term and real-time dynamic monitoring of bridge structures, because of the low degree of automation and the insufficient precision, causing bottlenecks and restriction. To solve this problem, we proposed a bridge displacement monitoring system based on laser projection-sensing technology. First, the laser spot recognition method was studied. Second, the software for the displacement monitoring system was developed. Finally, a series of experiments using this system were conducted, and the results show that such a system has high measurement accuracy and speed. We aim to develop a low-cost, high-accuracy and long-term monitoring method for bridge displacement based on these preliminary efforts.

## 1. Introduction

Bridges are an important part of the transportation infrastructure. Structural safety of bridges is not only related to the healthy and orderly development of the social, political, economic, national defense and other various undertakings of a country, but also directly affects the safety of the lives and properties of the people. Bridge displacement is the comprehensive reflection of the health status of a bridge structure and is the most important technical parameter in judging the overall vertical stiffness and bearing capacity of a bridge structure [1,2]. Bridge displacement monitoring is used to measure the displacement of each control section, thus providing a basis for the overall evaluation of the carrying capacity, operation status and endurance capability of a bridge structure. This measure can ensure the safe operation of a bridge structure and prolong its service life. By monitoring bridges, the defective fractures can be identified in a timely manner, thus reducing the repair and maintenance costs significantly and preventing the occurrence of bridge collapse and other major accidents [3,4,5].

Structural displacement monitoring can be accomplished by using numerous methods; the often-used methods are reviewed as follows. (1) Displacement sensors and acceleration sensors [6]: The displacement sensors are easy to use and relatively inexpensive, but in the case that the bridge is high, the displacement sensors cannot be installed under the bridge deck. Acceleration sensors have a negligible effect on structural vibration because of their small size and low weight. However, the identification effect of such sensors is poor for low-frequency static displacement. In addition, double integration is required to obtain the displacement; therefore, the accuracy of acceleration sensors is not high; (2) Laser Doppler vibrometer system [7]: The laser Doppler vibrometer system as a non-contact, non-destructive method of measuring the vibration and displacement of bridges has been shown to provide accurate results. However, this system is not suitable for long-term bridge monitoring, since it is oftentimes placed on the ground underneath the bridge and cannot be left unattended; (3) GPS satellite-surveying method [8,9]: The main advantages of this method are its applicability to all-weather continuous monitoring, as well as automatic completion of monitoring, recording and calculation. This high-technology method also has limitations in large-scale structural monitoring, such as the strict requirement of the antenna placement environment, low measurement rate, high cost and the need for highly skilled professionals; (4) Robotic total station [10,11]: This device has the advantages of high precision, automation and can measure the three-dimensional coordinates. This approach has been widely used in engineering displacement monitoring, but it cannot complete multi-target tasks in a short time. Moreover, due to its low measuring frequency, the requirement of dynamic measurement cannot be satisfied; (5) Terrestrial laser scanning [12,13]: This method can rapidly build complex, irregular 3D visualization scene models. However, the monitoring accuracy of each sampling point is low, and the post-processing of massive data is complicated; (6) Strain sensors: This method can draw the exact vertical displacement of a bridge, which, uniformly loaded on *n* + 1 supports with the use of 6 × *n* strain sensors, can also measure the deformation of the frame, and so on. However, it is unsuitable for measuring the mid-span deformation of long-span bridges [2,14].

In summary, the current use of a relatively wide range of monitoring methods for engineering structures may have some limitations that hinder the realization of the long-term and real-time dynamic monitoring for bridge displacement. In recent years, laser projection-sensing technology, the core technique of which is digital video processing, has been studied for object displacement monitoring. The above-mentioned monitoring method has many advantages [4]:

(1) High precision and low cost.
(2) High image acquisition frequency and able to reflect the variation of structural dynamic displacement completely.
(3) High image processing efficiency and able to meet the requirement of real-time collection, real-time processing and displacement curve real-time display.
(4) Meets the requirement of scalability analysis for the collected data.
(5) Easy to carry out technology upgrading; the digital form and transmission method of video can be easily combined with the existing digital communication and computer technology to provide people with new, more flexible, more convenient services.

Considerable research progress and several achievements have been attained by this technology in different fields.

In 2009, Zhang *et al.* [15,16] of China studied the laser projection-sensing technology and designed a practical bridge displacement monitoring experiment at He’ergou Bridge. Data were automatically collected 20 min/time, and the monitoring results were compared with the data of the water-level deflection instrument. The experiment showed that the laser projection method operated better and was more accurate than the water-level deflection instrument, especially for high-fall and long-span bridges. This experiment was followed by a study of the anti-interference capability and high precision laser spot location algorithm.

In 2010, Kanekawa and Matsuya, *et al.* [17] from Japan formed a relative-story displacement sensor with a laser device and a phototransistor (PT) array. When moved with the floor, the laser device projected the laser spot onto the PT array, and the relative displacement between the ceiling and the floor was estimated by the distribution of the PT output voltage. The accuracy of the measurements was 0.11 mm, with a dynamic range of 70 mm and with a sampling rate of 200 Hz.

In 2010, Myung *et al.* [18] from Korea proposed a visually-servoed paired structured light system, which was composed of two sides facing each other, each with a camera, a screen and one or two lasers controlled by a 2-DOF manipulator. In this system, the relative translational and rotational displacement between two sides can be estimated by calculating the positions of the projected laser beams on the screens and the rotation angles of the manipulators.

In 2011, Jeon *et al.* [19] studied a paired visual servoing system for 6-DOF displacement measurement of structures. In this study, a calibration method was proposed to offset the initial displacement using the first captured image, and a newly designed 2-DOF manipulator for each side was visually controlled to prevent the laser beams from leaving the screen.

In 2013, Thomas and Ali [20] proposed a methodology for a virtual visual sensor (VVS) to measure structural vibrations. The fundamental frequency of vibration of single-degree-of-freedom (SDOF) systems can be accurately computed using the proposed methodology of VVS, and multiple independently vibrating elements in one video can be distinguished and their fundamental frequency of vibration computed.

From the foregoing text, the object displacement monitoring method based on laser projection-sensing technology has gradually received considerable attention and has become widely used. Based on digital image processing techniques, the displacement monitoring software was developed to provide a real-time display of the two-dimensional coordinate values and the coordinate change curve of the laser spot. The hardware adopts commercially available optoelectronic devices and optical devices, which can be conveniently assembled, are low cost and are conducive to hardware equipment upgrading and expansion to a higher performance level. The experimental results show that this system is feasible for static and dynamic displacement monitoring.

## 2. Introduction of the Method

### 2.1. Basic Principle

**Figure 1:**
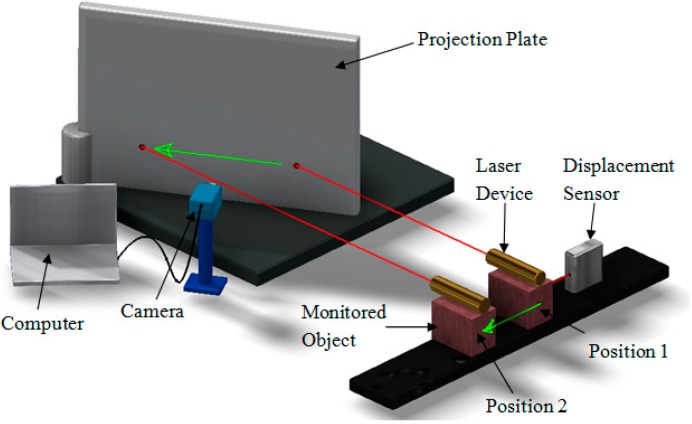
Experimental schematic diagram.

First, a laser device is mounted on the monitored object, making its optical path direction perpendicular to the movement direction of the object. Second, the angle between the projection plate plane and the laser optical path is adjusted to 30 degrees. During measurement, the industrial camera shoots the laser spot and outputs a video signal to the computer. Once the monitored object moves from Position 1 to Position 2, the laser device will follow the movement, such that the laser spot on the projection plate will move proportionally. The ratio (K) of the actual size to the pixel size, as well as the original position of the laser spot of the video signal are obtained through the computer program first, after which the video signal is processed to obtain the pixel coordinates (X) of the laser spot centroid of each frame image. Then, the actual coordinates of the laser spot centroid of each frame image can be obtained by the formula L = X × K, thereby visually reflecting the displacement of the monitored object. The experimental schematic diagram is shown in Figure 1.

### 2.2. Program Introduction

The program is based on MFC (Microsoft Foundation Classes) and developed in Microsoft Visual Studio 2010. The CV_BGR2GRAY function in OpenCV is employed for image gray processing, whereas the cvThreshold function is used for gray image binarization processing. The pixel coordinates of the laser spot centroid of the binarized image are obtained by the calculation formula for the centroid. To obtain the actual coordinates of the laser spot centroid, two steps are needed: calibration and acquisition.

#### 2.2.1. Calibration

**Figure 2:**
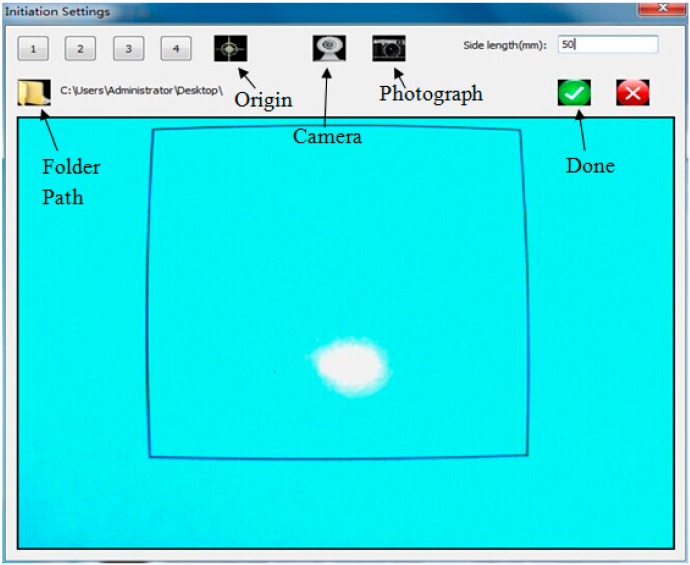
Program setup interface.

Print a square on white paper and attach the paper to the projection plate, making the laser spot located inside the square. Then, click the “setup” button in the main page of the “spot centroid acquisition” program. The setup interface is shown in Figure 2.

**Figure 3:**
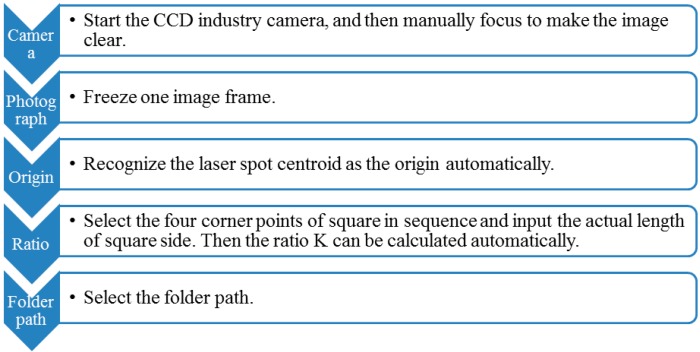
Calibration steps.

The calibration is divided into several minor steps, as shown in Figure 3. Each step corresponds to one or more buttons in the setup interface.

#### 2.2.2. Coordinate Acquisition

**Figure 4:**
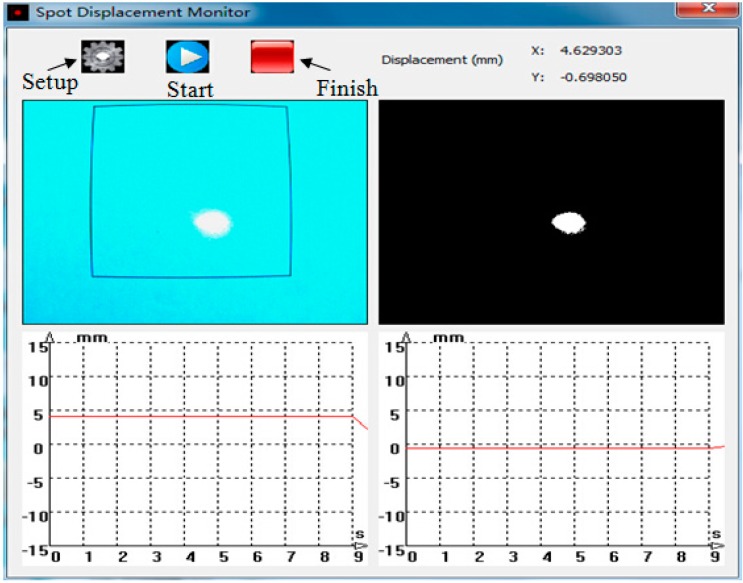
Main interface of the program.

After completing Step 1, the “done” button of the setup interface is clicked to return to the main interface of the program, as shown in Figure 4.

The main interface has four windows, namely the original video image, the binarized video image, as well as the laser spot real-time displacement curves of the X and Y directions. The top right side shows the real-time laser spot centroid displacement in the X and Y directions. In the binarized video image, the laser spot is white, whereas the background is black. When the “start” button is clicked, the program will start recording the centroid displacement of the white area, that is the laser spot centroid displacement. When the “finish” button is clicked, the program will save the coordinate values of the laser spot centroid into .txt file format.

As long as the laser spot within the scope of the camera vision can be identified, but if the camera is too far away from the projection plate, then the actual size of each pixel represented is too big, this will reduce the identification accuracy. The range of resolution that the actual size of each pixel represents is about 0.12 to 0.13 mm.

### 2.3. Data Processing

During the experiment, a laser displacement sensor was used to monitor the precise displacement of the object. When the projection plate plane and the optical path are angled at 30 degrees, by geometric relation, the displacement of the laser spot in the X direction is twice that when the projection plate plane and the optical path are angled at 90 degrees. After dividing the centroid displacement data of the laser spot by two, we can compare the result with the data of the laser displacement sensor. MATLAB was used to process the data collected by the program and the laser displacement sensor to obtain the centroid displacement curve image of the laser spot and the displacement curve image of the monitored object, respectively. By comparing these two images, the feasibility and accuracy of this system can be verified.

### 2.4. Increase in Identification Accuracy

**Figure 5:**
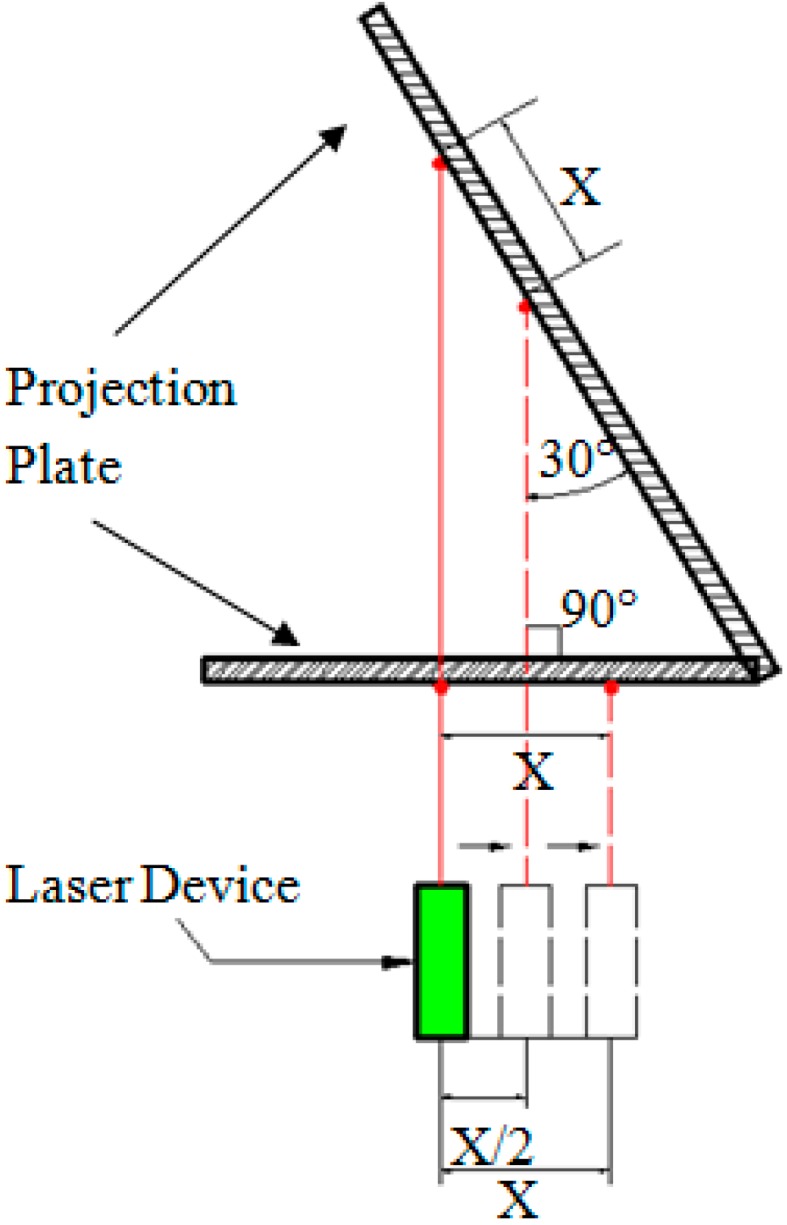
Schematic diagram of the increase in identification accuracy.

The actual size of each pixel represented is an important factor in determining identification accuracy. If the displacement change of the laser spot centroid between two adjacent image frames is within a single pixel, the program will be incapable of identifying the displacement change of the monitored object. Therefore, a smaller actual size of the pixel point represented results in a more sensitive identification of the displacement change of the object. Consider the following two cases, which are shown in Figure 5. In Case 1, the projection plate plane is perpendicular to the optical path. Assuming that when the object moves a distance of X in this case, the laser spot centroid in the video image just moves a distance of one pixel. In Case 2, the projection plate plane and the optical path are angled at 30°. In this case, when the object moves a distance of X/2, the laser spot centroid in the video image already moves a distance of one pixel. Therefore, the actual object displacement of each pixel represented in Case 2 is half of that in Case 1; thus, the identification accuracy is increased.

## 3. Experiments and Results

A series of static and dynamic displacement monitoring experiments have been conducted to prove the feasibility of the proposed program. The static displacement monitoring experiments aim to verify the stability of the proposed program. The dynamic displacement monitoring experiments are mainly used to observe whether the motion trail is successfully reflected when the monitored objected is moving quickly.

### 3.1. Main Instruments Parameters

The main instrument parameters are shown in Table 1.

**Table 1:** Main instrument parameters.

Instrument	Brand	Model	Parameter
Laser Device	Ruic	Red-light semiconductor laser	Output wavelength: 650 nm Overall dimensions: Φ 20 mm × 90 mm, Light spot diameter is 3 mm at a 20-m distance
Industrial Camera	Easyxin	Exin-U36	Resolution: 752 × 480 Frame rate: 40
Camera Lens	Easyxin	YX-0358	Focal length: 3.5 mm to 8 mm manual zoom
Laser Displacement Sensor	MICRO-EPSILON	ILD1300	Measuring range: 50 mm to 150 mm Resolution (dynamic): 100 microns

### 3.2. Static Experiment

The instruments used are the displacement loading device, laser device, industrial camera, white paper, projection plate, laptop computer and laser displacement sensor.

#### 3.2.1. Process of Static Experiment

**Figure 6:**
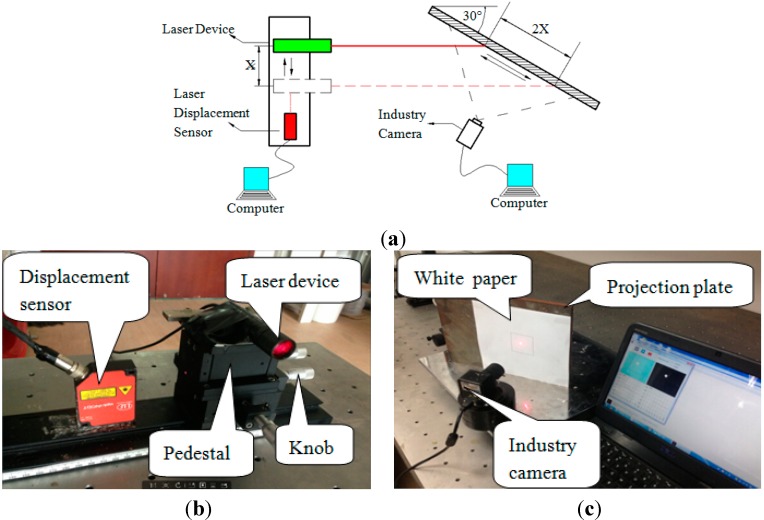
(**a**) Experimental schematic diagram of the static experiment; (**b**,**c**) experiment photos of the static experiment.

The laser device is mounted onto the pedestal of the displacement loading device. The knob of the pedestal is rotated to enable the pedestal to drive the laser device motion. The laser displacement sensor is adopted to measure the precise displacement of the pedestal. The displacement of the pedestal is 0.35 mm when the knob is rotated one complete lap. The loading process is so slow, that it can be assumed as static. The experimental schematic diagram and field experiment images are shown in Figure 6.

#### 3.2.2. Results of the Static Experiments

##### Simple Load Experiment

**Figure 7:**
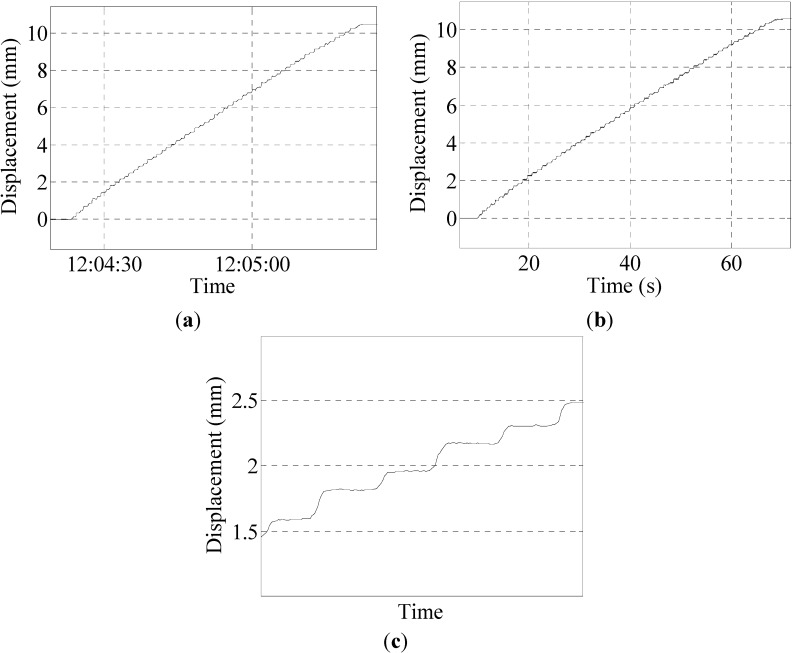
(**a**) Result of the program in Group 1; (**b**) result of the sensor in Group 1; (**c**) partial enlarged detail of the program result.

The knob was continuously rotated for 30 laps, and the total displacement of pedestal is about 10.5 mm. The results are shown in Figure 7.

As can be seen from the above figures, the peak datum of the laser displacement sensor is 10.58 mm, whereas that of the program is 10.54 mm. The difference is 0.04 mm, and the error between them is negligible at 0.38%.

##### Accuracy Testing Experiments

**Figure 8:**
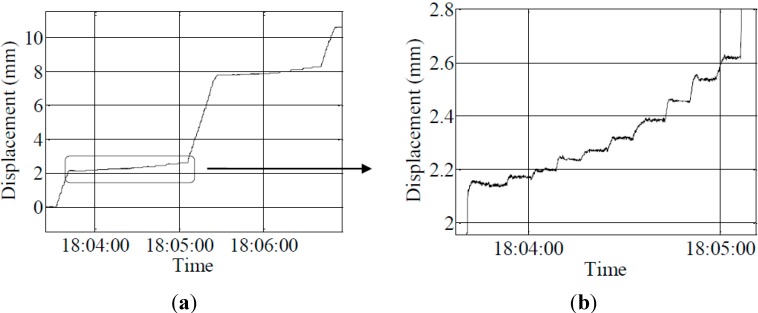
(**a**) Result of the program in Group 2; (**b**) partial enlarged detail of the program result.

Accuracy testing experiments aim to figure out whether the system can monitor the pedestal displacement change when the pedestal displacement is small. In the process of loading, the pedestal displacement is loaded from 0.01 mm to 0.02 mm, until 0.1 mm. The results are shown in Figure 8.

As shown in the figures above, when the displacement of the pedestal is 0.02 mm, the system can already reflect the change curve and the amount of change of the pedestal’s displacement. This proves that the system has high sensitivity, and the identification of small displacement is ideal.

##### Displacement Loop Experiment

**Figure 9:**
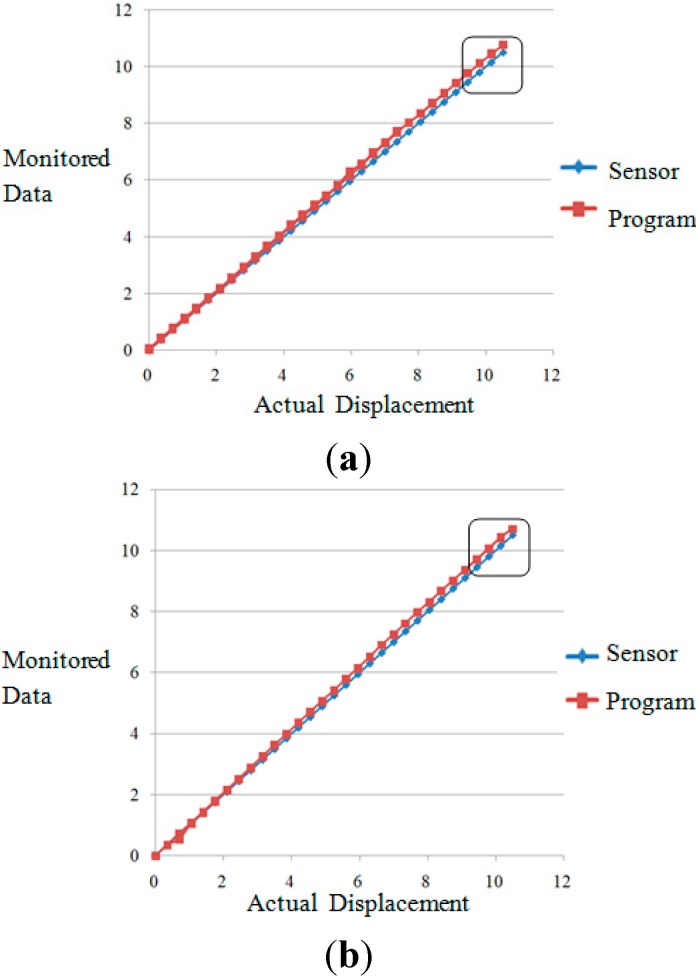

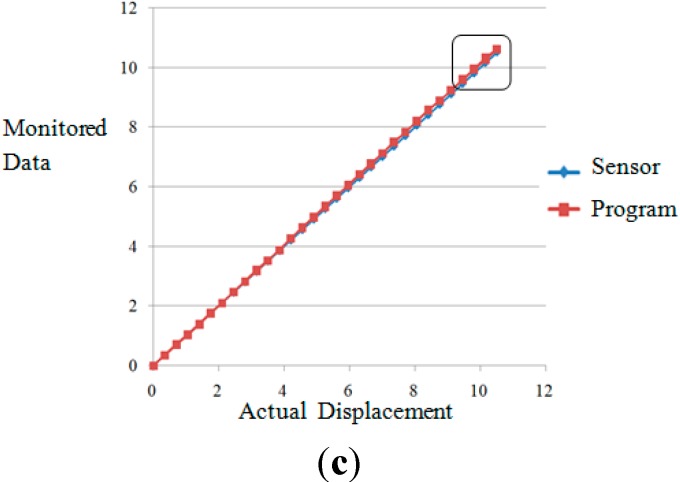
(**a**) First displacement loop; (**b**) second displacement loop; (**c**) third displacement loop.

The displacements of the laser spot centroid are manually recorded when the knob is rotated one lap. The total number of the rotations is 30, and the total displacement is approximately 10.5 mm. Loading is continuously applied three times back and forth, thus resulting in a three-loop process. The dotted lines shown in Figure 9 reflect the displacement of each loop, wherein the horizontal axis represents the displacement of the pedestal and the vertical axis represents the monitored displacement (the blue dotted line represents the data of the laser displacement sensor, whereas the red dotted line represents the data of the program).

The last four points of each loop were selected, seven data in total, and the data comparison of laser displacement sensor and the program is shown in Table 2.

**Table 2:** Comparison of the data of the sensor and program.

Program	9.760	10.110	10.455	10.765	10.455	10.110	9.755
Sensor	9.450	9.853	10.211	10.550	10.150	9.822	9.451
Difference	0.31	0.257	0.244	0.215	0.305	0.288	0.304
Error	3.28%	2.61%	2.39%	2.04%	3.00%	2.93%	3.22%
Program	9.715	10.065	10.425	10.715	10.405	10.05	9.695
Sensor	9.451	9.853	10.212	10.550	10.150	9.824	9.455
Difference	0.264	0.212	0.213	0.165	0.255	0.226	0.24
Error	2.79%	2.15%	2.09%	1.56%	2.51%	2.30%	2.54%
Program	9.610	9.960	10.335	10.620	10.275	9.93	9.585
Sensor	9.451	9.855	10.211	10.552	10.150	9.818	9.453
Difference	0.159	0.105	0.124	0.068	0.125	0.112	0.132
Error	1.68%	1.07%	1.21%	0.64%	1.23%	1.14%	1.40%

As shown in the figures above, the stability of the data of the program is very good; both are synthesized into a straight line, and the difference with the data of the laser displacement sensor is very small. The table above shows that the data error between the program and the laser displacement sensor is within 3.28%.

### 3.3. Dynamic Experiment of the Vibration Table

The instruments used are a vibration table, a laser device, an industrial camera, A4 paper, a projection plate, a laptop computer and a laser displacement sensor.

#### 3.3.1. Experiment Arrangement

The laser device is mounted on the vibration table and moves along with the vibration table. The laser displacement sensor is adopted to monitor the precise displacement of the vibration table.

**Figure 10:**
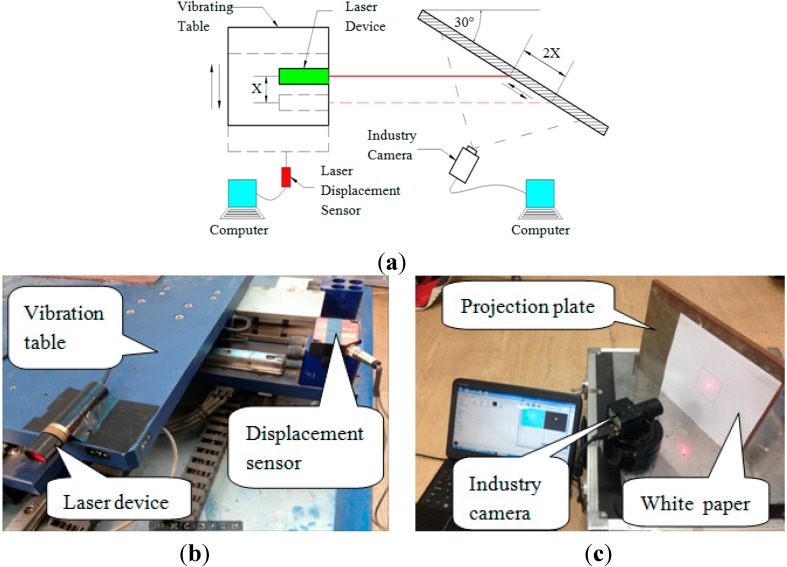
(**a**) Experimental schematic of the dynamic experiment; (**b**,**c**) field experiment of the dynamic experiment.

A series of dynamic experiments were performed, including experiments of three different seismic waves, each applied with three different incentives (0.2, 0.5 and 0.8), and experiments of sine waves of different amplitudes, namely, 10, 15 and 20 mm, each sine wave having a frequency change from 0.5 Hz, 1.0 Hz, 1.5 Hz to 5.0 Hz. The experimental schematic and images of the field experiments are shown in Figure 10.

#### 3.3.2. Experiment Results of the Vibration Table

The graphs show the results of the Japan Kobe earthquake wave; the incentive waves were 0.2 and 0.8, and for the results of the sine wave of 15-mm amplitude, the frequencies were 3.0 Hz, 3.5 Hz and 4.0 Hz.

**Figure 11:**
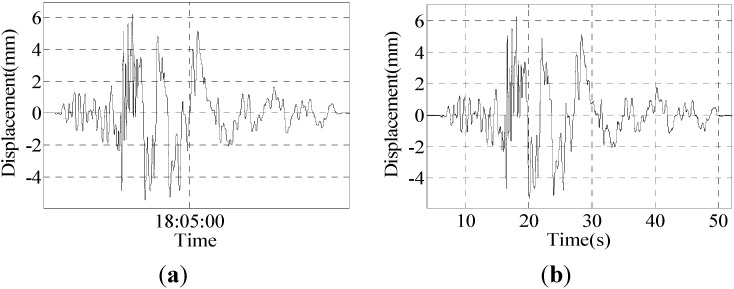

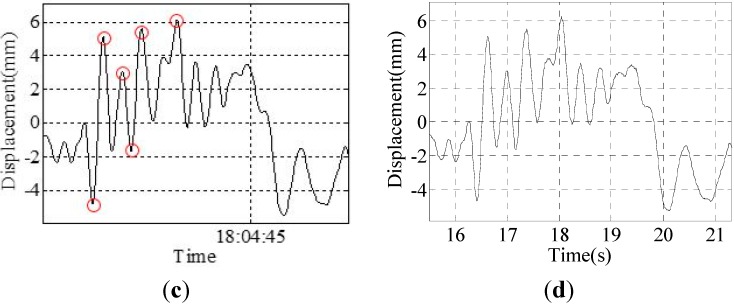
Results of Group 1: (**a**) result of the program; (**b**) result of the sensor; (**c**) partial enlarged detail of the result of the program; (**d**) partial enlarged detail of the result of the sensor.

Group I: The Japan Kobe earthquake of a 0.2 incentive wave and maximum displacement of about 6.2 mm; the results are shown in Figure 11.

Six peak point displacement values are selected to compare, and the results are shown in Table 3.

**Table 3:** Data comparison of the sensor and program.

Program	−4.853	5.142	3.086	−1.668	5.636	6.199
Sensor	−4.685	5.067	3.022	−1.648	5.517	6.242
Difference	0.168	0.075	0.064	0.02	0.119	0.043
Error	3.58%	1.48%	2.11%	1.21%	2.16%	0.69%

**Figure 12:**
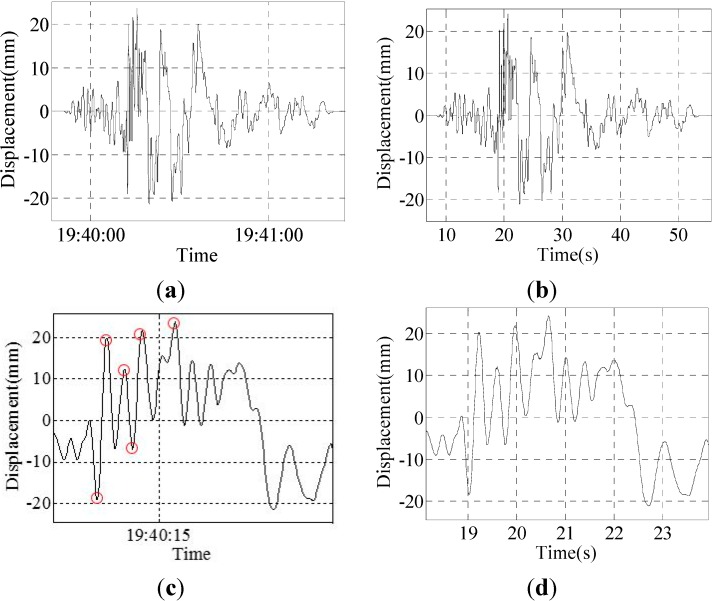
Results of Group 2: (**a**) result of the program; (**b**) result of the sensor; (**c**) partial enlarged detail of the result of the program; (**d**) partial enlarged detail of the result of the sensor.

Group II: The Japan Kobe earthquake of a 0.8 incentive wave and maximum displacement of approximately 24 mm; the results are shown in Figure 12.

Six peak point displacement values are selected to compare, and the results are shown in Table 4.

**Table 4:** Data comparison of the sensor and program.

Program	−19.000	19.970	12.39	−6.813	21.71	23.74
Sensor	−18.42	20.36	12.18	−6.53	21.94	24.09
Difference	0.58	0.39	0.19	0.283	0.23	0.35
Error	3.15%	1.91%	1.56%	4.33%	1.05%	1.45%

The experimental results show that when the random vibration of the vibration table occurs, the data differences between the program and laser displacement sensor are very small; the error is within 4.33%. The displacement change of the vibration table can be accurately and completely monitored by this system.

**Figure 13:**
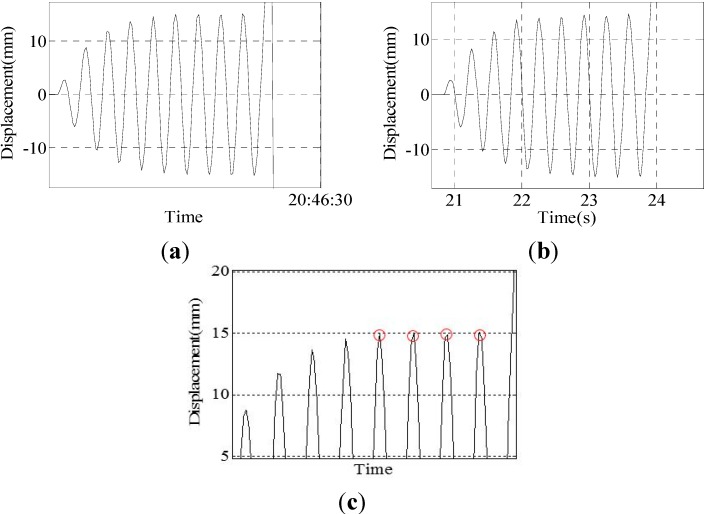
Results of Group 3: (**a**) result of the program; (**b**) result of the sensor; (**c**) peak curve of the result of the program.

Group III: The experimental results of the sine wave of a 3.0-Hz frequency and 15-mm amplitude are shown in Figure 13.

Four peak point displacement values are selected to compare, and the results are shown in Table 5.

**Table 5:** Data comparison of the sensor and program.

Program	14.95	15.01	14.88	15.10
Sensor	13.96	14.46	14.23	14.63
Difference	0.99	0.55	0.65	0.47
Error	7.10%	3.80%	4.57%	3.21%

**Figure 14:**
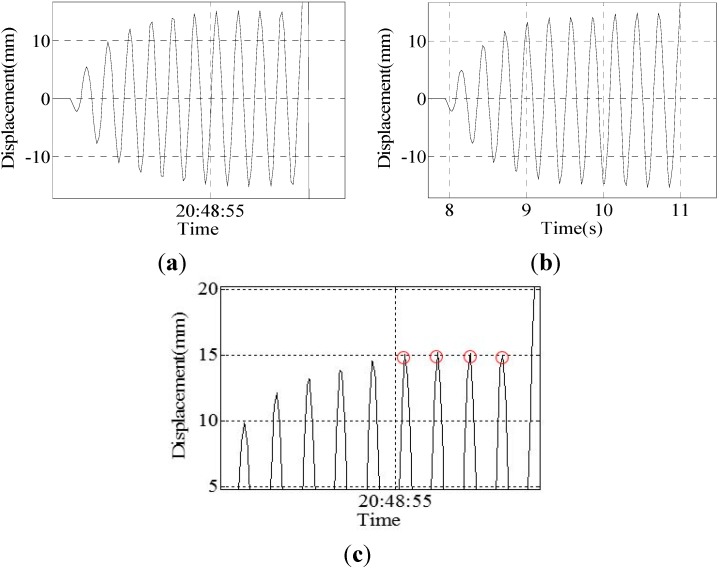
Results of Group 4: (**a**) result of the program; (**b**) result of the sensor; (**c**) peak curve of the result of the program.

Group IV: The experimental results of the sine wave of a 3.5-Hz frequency and 15-mm amplitude are shown in Figure 14.

Four peak point displacement values are selected to compare, and the results are shown in Table 6.

**Table 6:** Comparison of the data of the sensor and program.

Program	15.03	15.20	15.12	14.96
Sensor	14.09	14.66	14.83	14.77
Difference	0.94	0.54	0.29	0.19
Error	6.67%	3.68%	1.96%	1.29%

**Figure 15:**
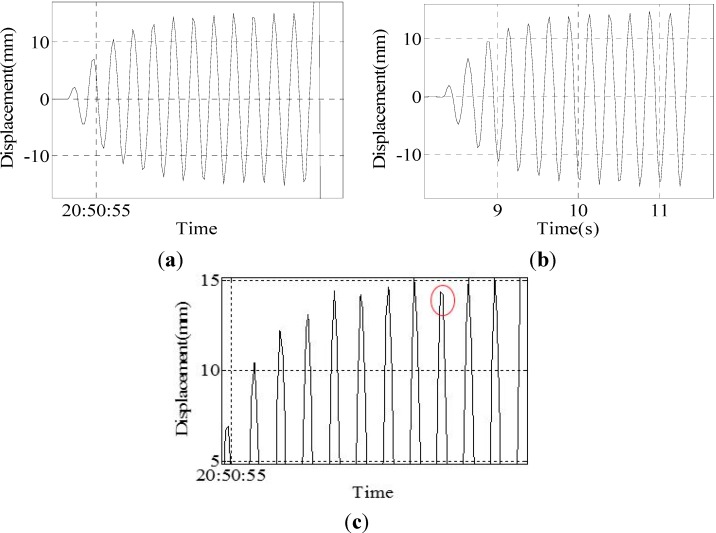
Results of Group 5: (**a**) result of the program; (**b**) result of the sensor; (**c**) peak curve of the result of the program.

Group V: The experimental results of the sine wave of a 4.0-Hz frequency and 15-mm amplitude are shown in Figure 15.

Due to the laser displacement sensor being placed next to the vibration table, the sensor will have a slight vibration caused by the vibration table; therefore, the accuracy of the monitoring will be decreased. It can be seen from Table 4 and Table 5 that the data of the laser displacement sensor are not ideal; the range of the peak points’ value is large, ranging between 13.96 and 14.83. However, the peak point data of the program is around 15 mm, ranging between 14.88 and 15.20, so the data of the program are better. The experimental results show that when the vibration frequency of the vibration table is less than or equal to 3.5 Hz, the displacement change of the vibration table can be accurately and completely monitored by this system. However, when the vibration frequency is greater than or equal to 4.0 Hz, some individual peak points of the displacement curve cannot be monitored. This limitation is attributed to the camera frame rate, which is approximately 40 frames per second. If the vibration is too fast, then the camera fails to capture all of the peak points, as shown in Figure 14c.

### 3.4. Dynamic Experiment of the Truss Bridge Model

The instruments used are a truss bridge model, a laser device, an industrial camera, A4 paper, a projection plate, a laptop computer and a laser displacement sensor. The model consists of 16-Mn steel, a bolt ball and a bolt. Each lever is 0.4 m long, and the model is 6.4 m long.

#### 3.4.1. Experiment Arrangement

**Figure 16:**
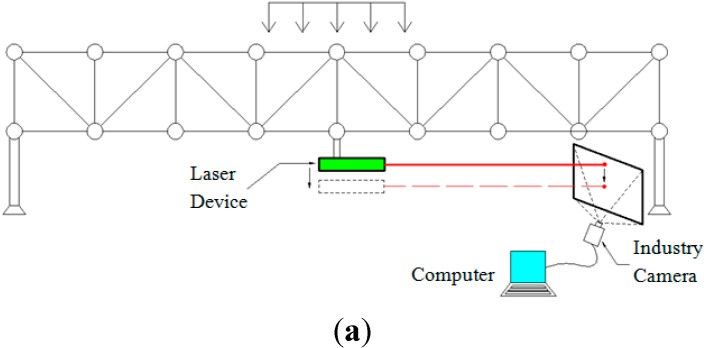

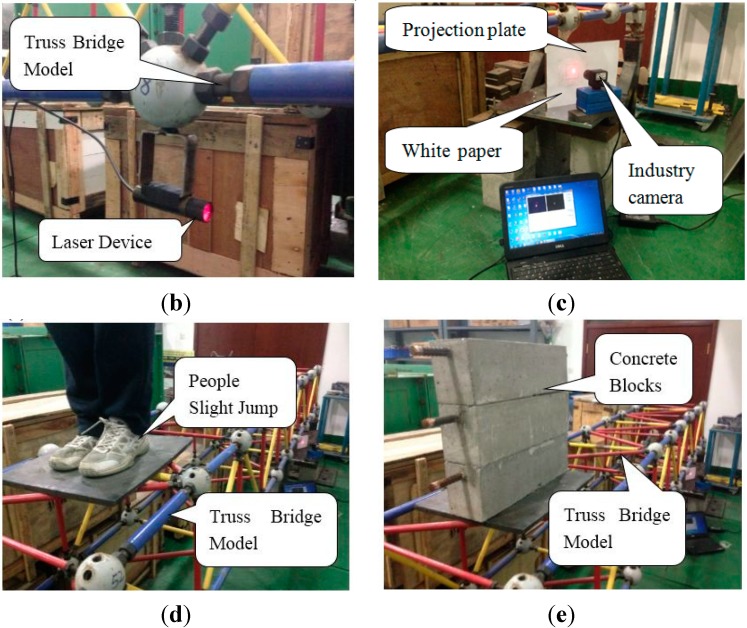
(**a**) Experimental schematic diagram of the static experiment; (**b–e**) photos of the static experiment.

The laser device is mounted on the bottom of the mid-span of the truss bridge model, and the projection plate is placed at one end of the model. The load is then applied to the mid-span of the model by people who are slightly jumping and laying concrete blocks. The experimental schematic and images of the field experiments are shown in Figure 16.

#### 3.4.2. Experiment Results

**Figure 17:**
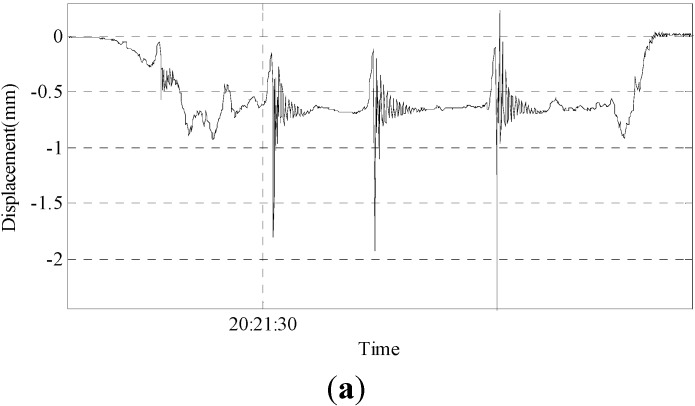

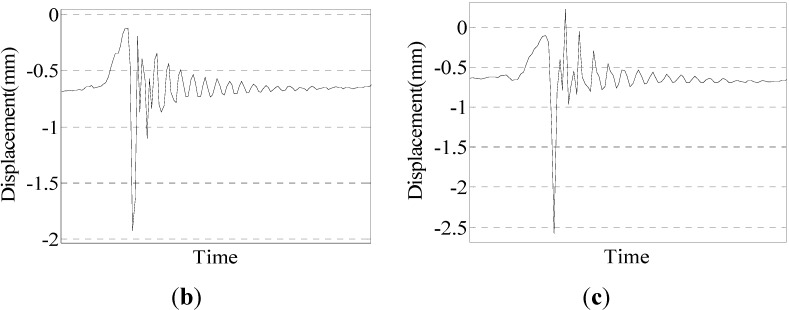
(**a**) Result of a person slightly jumping; (**b**) enlarged second vibration curve; (**c**) enlarged third vibration curve.

Group 1: The results of the experiment involving a 150-pound person slightly jumping in mid-span are shown in Figure 17.

As shown in the figures above, the maximum displacement is only approximately 2.6 mm, which is very small. However, this system can monitor the attenuation trend of the displacement well, and the monitoring result is ideal.

**Figure 18:**
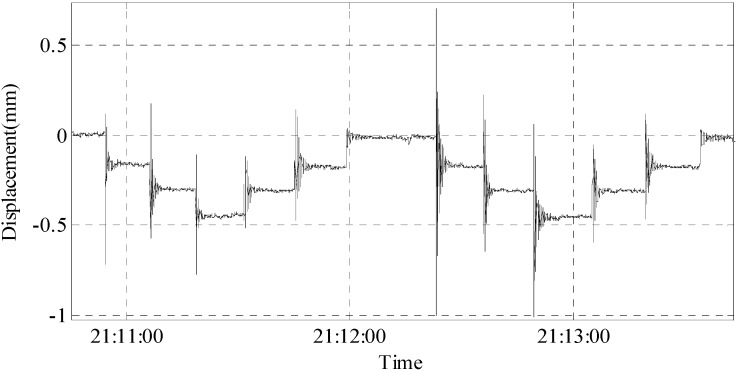
The result of the laying of concrete blocks.

Group 2: Three concrete blocks, each having a length of 50 cm and a cross-sectional area of 15 cm × 15 cm, are laid in the mid-span. Loading and unloading were repeated twice, and the experimental results are shown in Figure 18.

After each time of loading or unloading of a concrete block, the displacement change and vibration of the model can be clearly detected. The displacement changes are shown in the Table 7.

**Table 7:** Displacement changes of the model.

First Cycle	0	−0.1611	−0.3014	−0.4448	−0.3056	−0.1708	−0.0097
Difference (mm)	-	0.1611	0.1403	0.1413	0.1392	0.1348	0.1611
Second cycle	−0.0097	−0.1726	−0.3072	−0.4501	−0.3085	−0.1749	−0.0147
Difference (mm)	-	0.1629	0.1346	0.1429	0.1416	0.1336	0.1602

As shown in Table 6, after loading the first piece or unloading the last piece of the concrete block, the model displacement is approximately 0.1610 mm. In other cases, the model displacement is approximately 0.1400 mm.

## 4. Conclusions

Through a series of experiments and the analyses of the experimental data, it can be concluded that the precision of the displacement monitoring system based on the laser projection-sensing technology is ideal. In the static experiments, when the displacement is 10.50 mm, the error is 0.04 mm and the recognition of the minimum distance change is 0.02 mm. The experiments of the vibration table can reflect the variation of displacement completely and accurately. The experiments of the steel girder bridge model can monitor the attenuation trend of displacement well, and the displacement repeatability is likewise very good. These results show that the system has the advantages of a simple structure, high accuracy, low cost, ease of operation and stable performance. The system can be a feasible method to monitor bridge displacement. We believe that in the future, with the use of long-distance laser devices with a higher power and a better collimation, large-span bridges and other large structures’ displacement monitoring can be achieved, and with the use of cameras with a higher number of pixels and a higher frame rate, the monitoring of a wider range of displacements and faster vibration of the monitored objects can be realized.
